# *In Vivo* Time-Resolved Microtomography Reveals the Mechanics of the Blowfly Flight Motor

**DOI:** 10.1371/journal.pbio.1001823

**Published:** 2014-03-25

**Authors:** Simon M. Walker, Daniel A. Schwyn, Rajmund Mokso, Martina Wicklein, Tonya Müller, Michael Doube, Marco Stampanoni, Holger G. Krapp, Graham K. Taylor

**Affiliations:** 1 Department of Zoology, University of Oxford, Oxford, United Kingdom; 2 Department of Bioengineering, Imperial College London, London, United Kingdom; 3 Swiss Light Source, Paul Scherrer Institute, Villigen, Switzerland; 4 Department of Comparative Biomedical Sciences, The Royal Veterinary College, London, United Kingdom; 5 Institute for Biomedical Engineering, ETH Zurich and University of Zurich, Switzerland; Lund University, Sweden

## Abstract

Dipteran flies are amongst the smallest and most agile of flying animals. Their wings are driven indirectly by large power muscles, which cause cyclical deformations of the thorax that are amplified through the intricate wing hinge. Asymmetric flight manoeuvres are controlled by 13 pairs of steering muscles acting directly on the wing articulations. Collectively the steering muscles account for <3% of total flight muscle mass, raising the question of how they can modulate the vastly greater output of the power muscles during manoeuvres. Here we present the results of a synchrotron-based study performing micrometre-resolution, time-resolved microtomography on the 145 Hz wingbeat of blowflies. These data represent the first four-dimensional visualizations of an organism's internal movements on sub-millisecond and micrometre scales. This technique allows us to visualize and measure the three-dimensional movements of five of the largest steering muscles, and to place these in the context of the deforming thoracic mechanism that the muscles actuate. Our visualizations show that the steering muscles operate through a diverse range of nonlinear mechanisms, revealing several unexpected features that could not have been identified using any other technique. The tendons of some steering muscles buckle on every wingbeat to accommodate high amplitude movements of the wing hinge. Other steering muscles absorb kinetic energy from an oscillating control linkage, which rotates at low wingbeat amplitude but translates at high wingbeat amplitude. Kinetic energy is distributed differently in these two modes of oscillation, which may play a role in asymmetric power management during flight control. Structural flexibility is known to be important to the aerodynamic efficiency of insect wings, and to the function of their indirect power muscles. We show that it is integral also to the operation of the steering muscles, and so to the functional flexibility of the insect flight motor.

## Abstract 2

Time-resolved X-ray microtomography permits a real-time view of the blowfly in flight at a previously unprecedented level of detail, revealing how the tiny steering muscles work.

## Author Summary

A blowfly's wingbeat is 50 times shorter than a blink of a human eye, and is controlled by numerous tiny steering muscles—some of which are as thin as a human hair. To visualize the movements of these muscles and the deformations of the surrounding exoskeleton, we developed a technique to allow us to look inside the insects during tethered flight. We used a particle accelerator to record high-speed X-ray images of the flying blowflies, which we used to reconstruct three-dimensional tomograms of their flight motor at ten different stages of the wingbeat. We measured the asymmetric movements of the steering muscles associated with turning flight, together with the accompanying movements of the wing hinge—arguably the most complex joint in nature. The steering muscles represent <3% of total flight muscle mass, so a key question has been how they can modulate the output of the much larger power muscles. We show that by shifting the flight motor between different modes of oscillation, the fly is able to divert mechanical energy into a steering muscle that is specialized to absorb mechanical energy. In general, we find that deformations of the muscles and thorax are key to understanding this remarkable mechanism.

## Introduction

Insects are the smallest and most agile of all flying animals. These attributes are taken to extremes in the dipteran flies, whose single pair of wings enable a range of dramatic flight manoeuvres, from turning on the spot, or flying backwards, to even landing on ceilings. The blowfly *Calliphora vicina* routinely pulls up to four times its body weight during turns [1], and its seemingly simple reciprocal wingbeat belies the complexity of the flight motor that drives it [2]. Each of the two wings is powered by four stretch-activated muscles that undergo self-induced oscillations at a frequency in excess of 100 Hz. Rather than attaching directly to the wings, these indirect power muscles drive small amplitude deformations of the thorax, which are then amplified through the intricate wing hinge [3]. This arrangement leaves little scope for the indirect power muscles to create the wing kinematic asymmetries that are required for asymmetric flight manoeuvres [4],[5]. Instead, kinematic asymmetries are produced by the 13 steering muscles [6],[7]. Collectively the steering muscles have <3% of the total mass of the indirect power muscles, which leads to a key, unresolved question. How are the tiny steering muscles able to shape the vastly greater—and essentially symmetric—output of the indirect power muscles [4],[5], so as to produce the large wingbeat asymmetries that enable fast flight manoeuvres?

The wing articulates with the thorax through a complex arrangement of cuticular structures called sclerites, which the steering muscles actuate [3],[6],[7]. Here, we provide a brief overview of the sclerites and associated muscles. A more detailed anatomical description of the muscles and their attachment points in blowflies can be found in [6], and these are described for the dipteran flight motor in general in [3]. The wing hinge is formed by four axillary sclerites, but only the first, third, and fourth of these have steering muscles attached. In flies, the fourth axillary sclerite is fused to the thoracic wall, and is usually referred to as the posterior notal wing process. The wing base is also connected by ligaments to the external head of a lever-like control linkage called the basalare sclerite, which projects into the thorax. In summary, ten steering muscles insert on the axillary sclerites; a further three insert on the basalare sclerite [3],[6]. Rather little is known about how the steering muscles modify the motions of these sclerites, but electrophysiological studies have correlated the activation states of some of these muscles with variation in wingtip kinematics, principally focussing upon variation in stroke amplitude. For example, during visually stimulated roll responses in *Calliphora*, activity of the first and second basalare muscles, and activity of at least some of the third axillary muscles, is associated with increased stroke amplitude. However, because several steering muscles are active during roll manoeuvres [3],[8],[9], the individual function of each muscle cannot readily be inferred from electrophysiological and wing kinematic data alone. Other work has attempted to identify the effects of different groups of steering muscles upon the aerodynamic forces and moments [10], but the specific mechanisms through which the steering muscles manipulate the wing hinge sclerites remain elusive.

**Figure 1:**
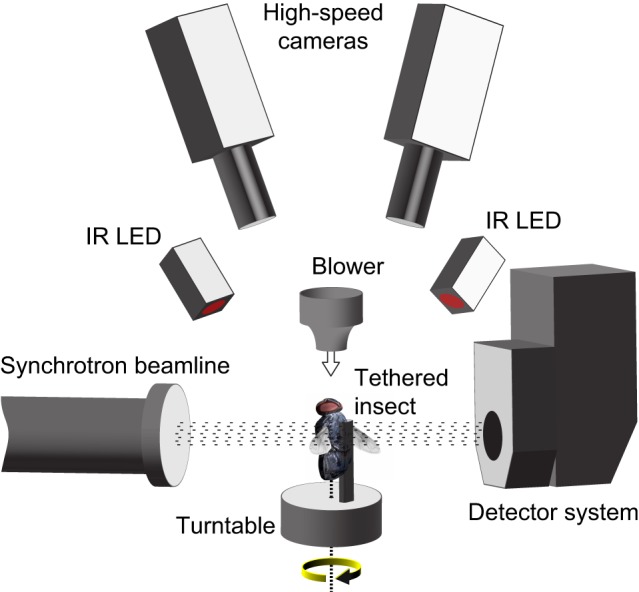
Schematic diagram of the experimental setup, showing the direction of the wind stimuli (white arrow) and rotational stimuli (yellow arrow).

Elucidating muscle function fully requires measurements of stress, strain, and activation, combined with knowledge of the mechanism the muscle actuates [11]. These measurements can be made simultaneously in larger vertebrates [12], but this has not yet been achieved in insects. Most of our current understanding of steering muscle function comes from anatomical [6],[7],[13] and electrophysiological [8],[9],[14]–[19] studies, and we know surprisingly little about the mechanics of how the steering muscles control the wingbeat. This is due in part to the extraordinary difficulty of measuring micrometre-scale muscle movements *in vivo* at frequencies in excess of 100 Hz. Indeed, although patterns of muscle activation [4],[8]–[10],[14],[16]–[19] and stresses produced under work-loop conditions [20] have been characterised for some insect steering muscles, almost nothing is known about the associated muscle strains and the resulting thoracic movements. Techniques used to measure and visualize muscle strains in vertebrates, such as sonomicrometry [21],[22] and stereo X-ray imaging [23], are unsuited to insects. Strains have been measured in insect power muscles using vivisective microscopy [24], external markers [25], and X-ray diffraction [26], but the smaller size of the steering muscles and their close interaction with the wing hinge makes them inaccessible even to these methods. To study the kinematics of the steering muscles, we therefore developed a new imaging technique allowing high-resolution, time-resolved microtomography of blowflies (*C. vicina*) in tethered flight (Figure 1).

Microtomography has previously been used *in vivo* to make time-resolved measurements of mouse hearts and lungs [27],[28], but to resolve the actuation of the insect flight motor we have extended the spatial and temporal resolutions of the technique by an order of magnitude each. This allowed us to produce tomographic visualizations of the instantaneous state of the flight motor for ten evenly spaced phases of the wingbeat (Movie S1, view here). We used these data to measure and compare the muscle strains and thoracic movements associated with different wingbeat kinematics. Taken together, our results emphasise the importance of muscular and cuticular deformations in modulating and controlling the kinematics of flapping flight.

### Methods Summary

We undertook time-resolved microtomographic imaging of the thorax of tethered blowflies flying in the TOMCAT beamline of the Swiss Light Source [29]. We used single exposure phase retrieval to increase contrast by an order of magnitude over standard absorption-based imaging [30]. This was important to enable the high acquisition rates and short exposure times required to resolve the wingbeat cycle. The insects were tethered to a rotating stage that underwent four complete revolutions per recording, thereby allowing radiographs to be taken from multiple evenly spaced viewing angles whilst the insect was flying (Figure 1). We simultaneously captured the three-dimensional wingtip kinematics using stereo high-speed photogrammetry [31] and grouped the radiographs according to the wingtip position. Each group contained multiple radiographs corresponding to the same phase of the wingbeat, but taken from different viewing angles. This allowed us to reconstruct tomograms for each group separately, producing tomograms for ten evenly spaced phases of the wingbeat. Each tomogram pools radiographs from *c*. 600 wingbeats and therefore represents the average state of the flight motor at the corresponding phase of the wingbeat.

### Results and Discussion

**Figure 2:**
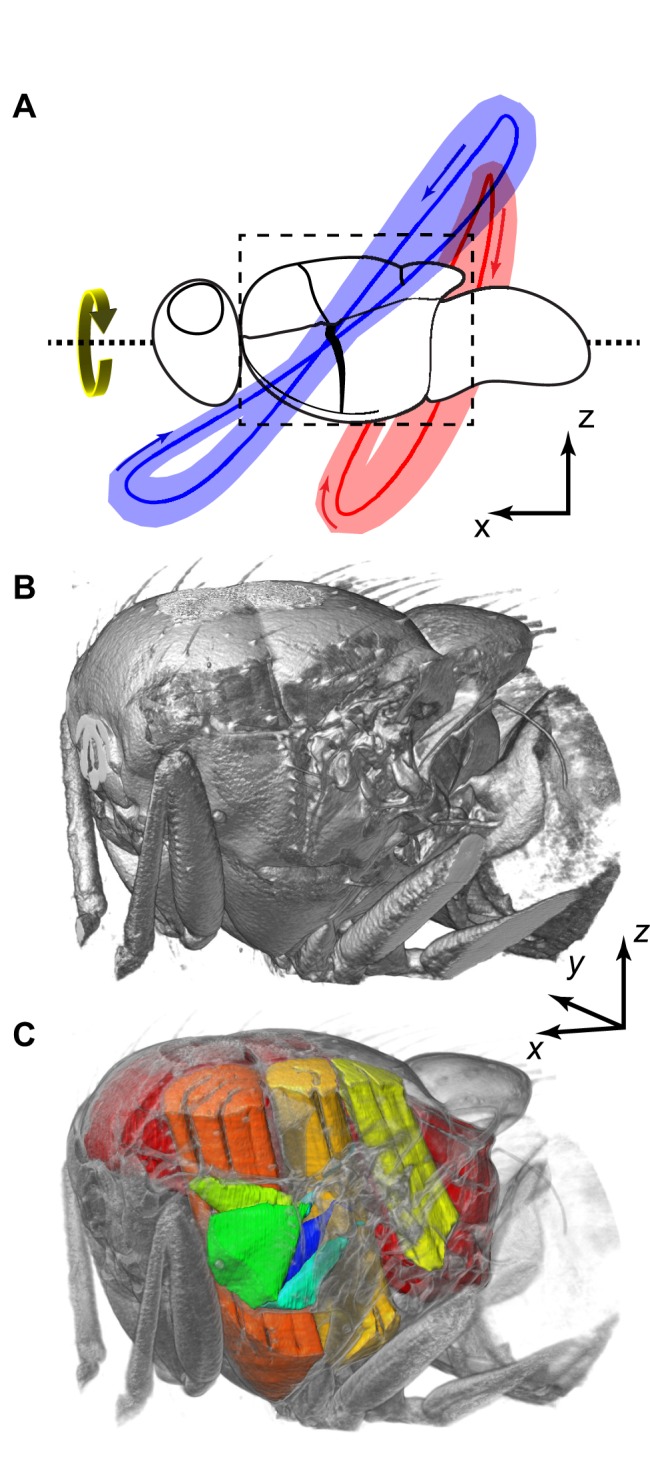
Overview. (A) Mean (red/blue lines) and standard deviation (red/blue shading) of wing tip position through all of the wingbeats of all four flies, showing differences in wing tip path between the left, high-amplitude (blue) and right, low-amplitude (red) wings. The arrows indicate the direction of the wings' movement. (B) External visualization of the thorax, covering the region outlined in (A). (C) Cutaway visualization of the thorax showing the five steering muscles analysed (green to blue) and the power muscles (yellow to red). Movie S1 provides an animated overview of the movements of these muscles (view Movie S1 here).

**Figure 3:**
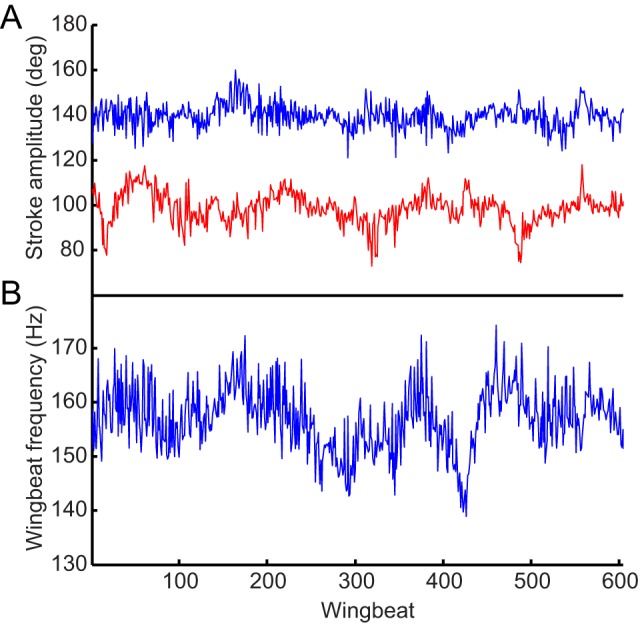
Wing kinematic parameters for one fly over entire recording period. (A) Stroke amplitude for the left (blue) and right (red) wings. (B) Wingbeat frequency, calculated as the mean of both wings.

**Figure 4:**
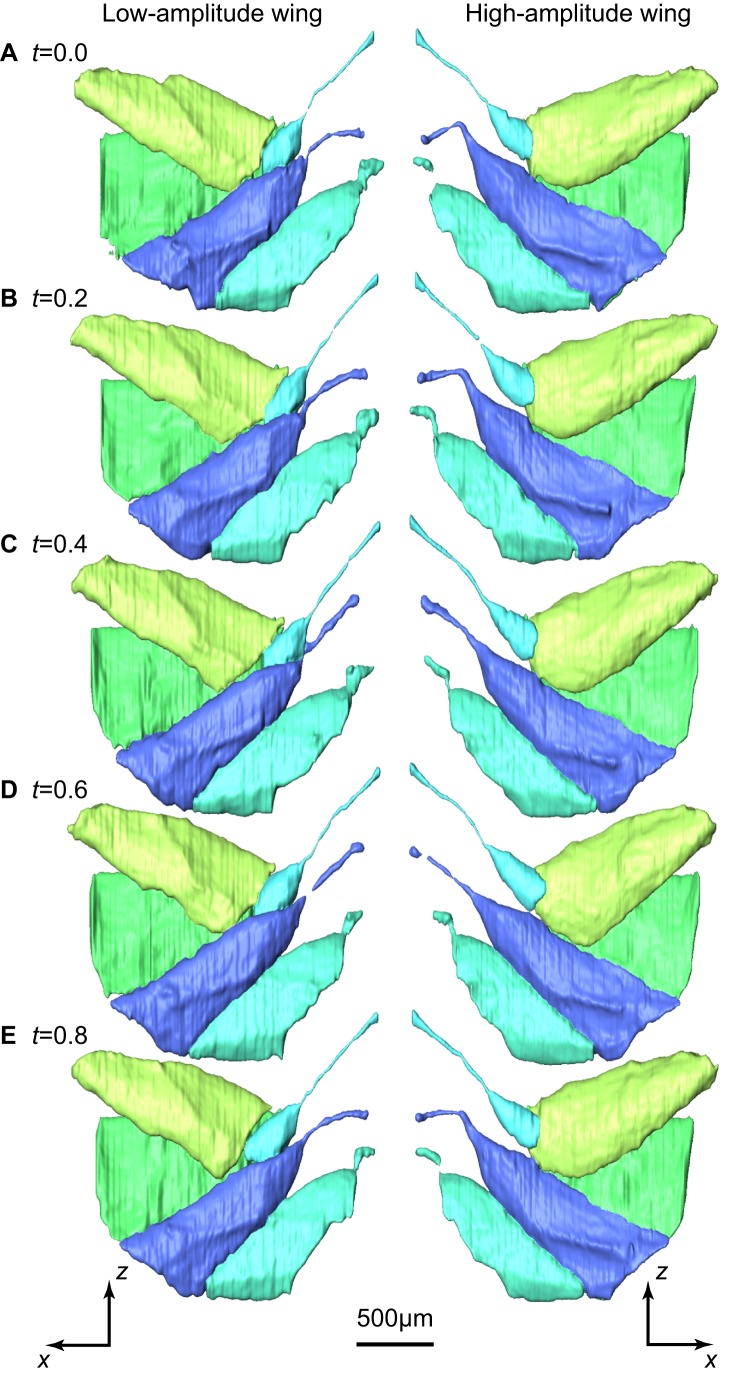
Three-dimensional surface renderings of five of the direct steering muscles in the low-amplitude (left column) and high-amplitude (right column) wings. Five of the ten stages of the wingbeat cycle are shown for one individual, starting at the beginning of the downstroke. The times (*t*) marked on each panel denote the proportion of the time through the wingbeat cycle. The steering muscles are viewed from the inside of the thorax looking out toward the wing hinge, and other parts of the thorax have been removed for clarity. See main text and Figure 6 for labeling of muscles, which follows the same colour scheme. Note the asymmetries in the buckling of the I1 tendon (dark blue) at the start of the stroke. The asymmetries in the movements of the other steering muscle are almost imperceptible in this figure, but they are clearly visible in the accompanying animation of all ten stages of the wingbeat cycle in Movie S2 (view Movie S2 here). The muscles and tendons were segmented manually. Note, however, that the small diameter and fast movement of the tendons leads to occasional data dropout (e.g., *I1* tendon at *t* = 0.6).

**Figure 5:**
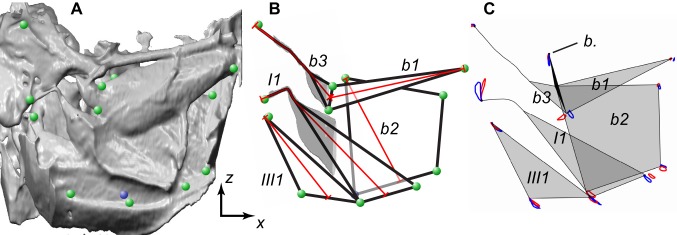
Measurements of muscle strains. (A) Three-dimensional surface rendering showing the internal view of the left steering muscles (high-amplitude wing). The steering muscles are viewed from the inside of the thorax looking out toward the wing hinge. The green circles indicate the endpoints of the muscles that were tracked. The blue circle shows the ventral base of *b2*, which is hidden from view behind the *I1* and *III1* muscles. A clipping plane was used to remove these muscles so that the base of *b2* was visible and could be tracked. (B) Schematic showing approximate shape of the five steering muscles (black lines) and the lines along which the muscle lengths were calculated (red lines). The grey shaded regions of *I1* and *b3* show where 3D skeletonization was used to find the centre line of the tendons to take buckling into account. (C) Diagram showing the movements of the endpoints of the steering muscles for the high-amplitude (blue orbits) and low-amplitude (red orbits) wings, averaged across flies. The view shown here corresponds to that in (C), with data for the other wing mirrored about the sagittal plane and overlain. The schematic representations of the muscles (shaded grey) and tendons (black lines) indicate the mean posture of the muscles at the start of the downstroke. *b*., basalare sclerite (filled black).

The flies were rotated during radiographic acquisition (332° s^−1^ or 347° s^−1^), producing a left-handed visual and inertial roll stimulus in the brightly lit lab environment (Figures 1, 2A, and 3). The left wing had consistently higher stroke amplitude than the right wing (141±7° *versus* 100±9°; mean ± standard deviation), and a shallower stroke plane (47±4° *versus* 68±10°), typical of a stabilizing roll response [13],[19],[32],[33]. The results of our experiments therefore allow us to compare the muscle strains and thoracic movements associated with simultaneous high versus low amplitude wingbeats in each individual. We analysed all three muscles inserting on the basalare sclerite (*b1*, *b2*, *b3*), and the two largest muscles (*I1*, *III1*) inserting on the first and third axillary sclerites (Figures 4 and 5). Together, these make up most of the mass of the steering muscles [6],[7]; the other eight steering muscles are smaller and could not be distinguished reliably from the surrounding tissues.

**Figure 6:**
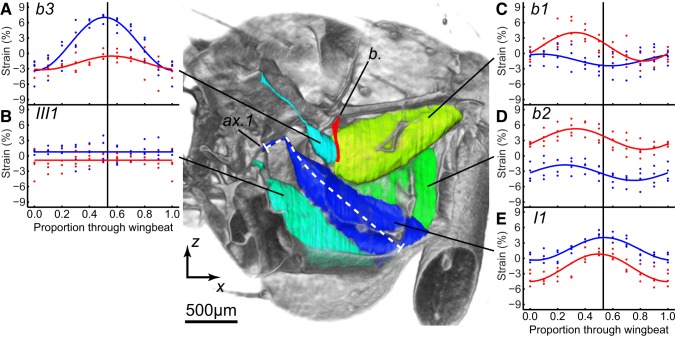
Cutaway visualization of the steering muscles and measured strains. Cutaway visualization of the steering muscles, looking out toward the wing hinge, with graphs of their measured strains (blue, high-amplitude wing; red, low-amplitude wing). The data points plot the strains measured for each individual fly (*n* = 4) at every stage of the wingbeat, starting at the beginning of the downstroke. The fitted curves are simple harmonic functions, except in the case of *III1*, which showed no significant time-periodic strain. Black vertical lines indicate the mean timing of the start of the upstroke. The strain in *I1* was measured along a line running down the middle of the muscle and its tendon (white dashed line) to take account of buckling. Movie S2 animates this view through the wingbeat for both wings. *b*., basalare sclerite (filled red); *ax.1*, first axillary sclerite. Movie S2 can be viewed here.

**Figure 7:**
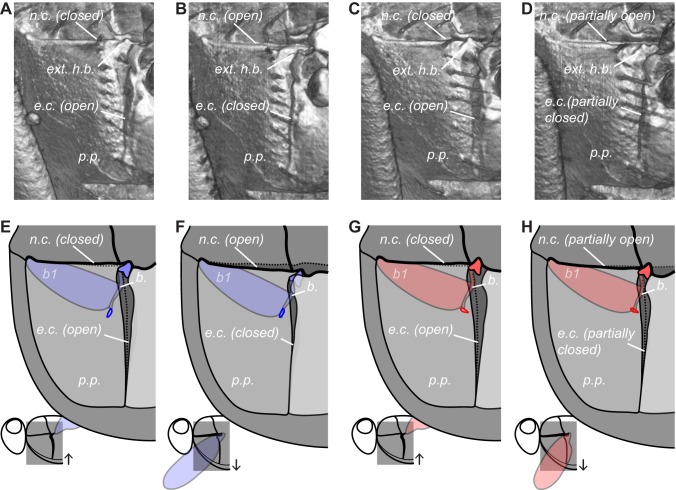
Movements of the basalare sclerite and pleural plate. External visualizations (A–D) and schematics (E–H) of the section of thorax indicated on the inset diagrams for the high-amplitude wing (A,B,E,F) and the low-amplitude wing (C,D,G,H). (A,C,E,G) Start of the downstroke. (B,D,F,H) Start of the upstroke. The pleural plate rotates as the thorax deforms through the wingbeat, causing oscillations of the basalare sclerite, which articulates with it. The small blue (high-amplitude) and red (low-amplitude) loops in (E–H) plot the path of the tip of the internal arm of the basalare sclerite, which oscillates rotationally on the low-amplitude wing and translationally on the high-amplitude wing. Movie S3 animates the external views of these movements through the wingbeat. *b*., basalare sclerite; *b1*, first muscle of the basalare sclerite; *e.c*., episternal cleft; *ext. h.b*., external head of the basalare sclerite; *n.c*., notopleural cleft; *p.p*., pleural plate. Movie S3 can be viewed here.

We first used our visualizations to describe the motions of the thoracic mechanisms that the steering muscles actuate (Movie S1, view here; Movie S2, view here; Movie S3, view here). The muscles that attach to the first axillary sclerite insert on its internal arm, which projects into the thorax and moves in opposition to the wing [7]; in contrast, the third axillary sclerite moves rather little relative to the base of the thorax (Movie S2, view here). The lever-like internal arm of the basalare sclerite oscillates back-and-forth (Figure 6; Movie S2, view here), while its external head articulates with a moving part of the thoracic wall called the pleural plate (Figure 7; Movie S3, view here). This hardened region of thoracic wall swings antero-ventrally on the downstroke, accommodated by the alternate opening and closing of two orthogonal clefts at its borders [34]. Rotation of the pleural plate was clearly responsible for driving oscillations of the basalare sclerite, which were of greater amplitude on the high-amplitude wing (Movie S3, view here).

**Figure 8:**
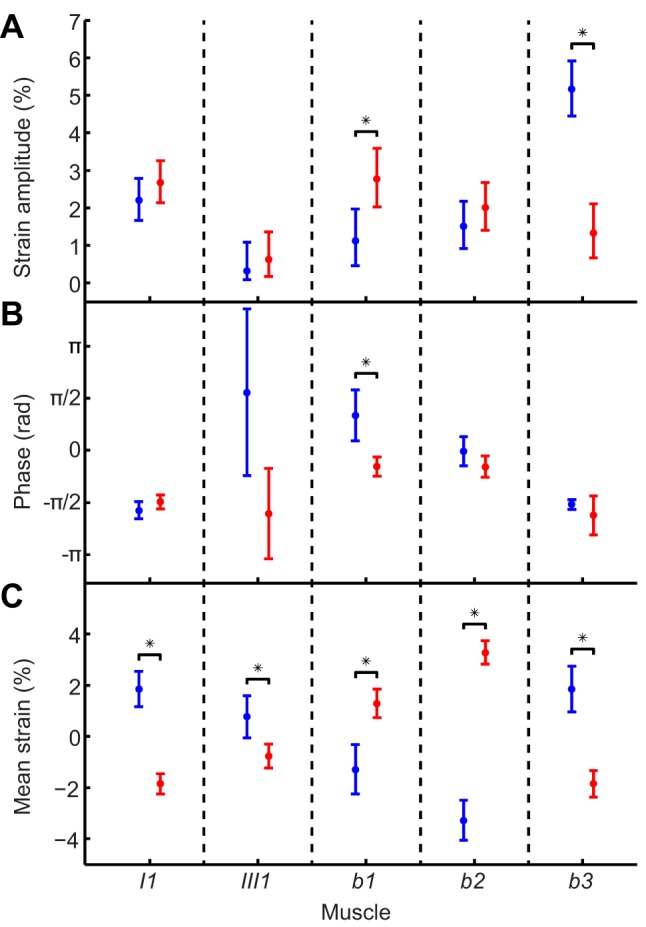
Statistical analysis of steering muscle asymmetries. 95% confidence intervals computed for: (A) amplitude of oscillatory muscle strain, (B) phase of oscillatory muscle strain, relative to start of the downstroke; (C) mean muscle strain. Vertical bars denote 95% confidence intervals; points denote actual parameter estimate. Non-overlapping 95% confidence intervals are starred to indicate the statistically significant differences between the high-amplitude (blue) and low-amplitude (red) wings.

The wingbeat asymmetries that we measured were associated with bilateral asymmetries in steering muscle kinematics (Figures 4–6; Movie S2, view here), which we quantified by measuring strains directly from the tomograms (Figures 5, 6, and 8). We were unable to measure muscle resting length for the purposes of normalizing muscle strains because the flies were flying continuously and reacted to the roll stimulus throughout each recording. Instead, we referenced the strain of each muscle in a pair to the pooled mean length of both muscles, which allowed us to compare muscle strains within each pair and between flies. Mean muscle strain was bilaterally asymmetric within each muscle pair (Figure 8C): higher on the high-amplitude wing for muscles *I1*, *III1*, and *b3*; but lower on the high-amplitude wing for muscles *b1* and *b2* (Figure 6). All of the muscles except *III1* displayed detectable strain oscillations at wingbeat frequency, but we could only detect statistically significant bilateral amplitude asymmetries in muscles *b1* and *b3* (Figure 8A). The amplitude of these strain oscillations was twice as high on the low-amplitude wing for *b1* (Figures 6C and 8A), and four times as high on the high-amplitude wing for *b3* (Figures 6A and 8A). The *b1* strain oscillations also displayed a statistically significant phase asymmetry, with the oscillations on the low-amplitude wing delayed by a quarter of a wingbeat (Figure 8B).

Muscle strains need not always be caused by contraction of the muscle itself. For example, work-loop measurements have shown that *b1* is specialized to do negative work (i.e., to absorb rather than impart kinetic energy), and is unable to cycle fast enough to drive oscillations at wingbeat frequency [20]. The measured *b1* oscillations must therefore have been driven by oscillations of the basalare sclerite forced by movement of the wing and thorax (Movie S1, view here; Movie S2, view here; Movie S3, view here). We cannot say unequivocally why the *b1* strain oscillations were bilaterally asymmetric, but in principle this must reflect either asymmetric loading or asymmetric stiffness. Electrophysiological studies have shown that *b1* is activated earlier with increasing wingbeat amplitude, which increases both its stiffness and the amount of negative work done under a given strain [9],[10],[19]. It has therefore been hypothesised that this increased stiffness should cause the amplitude of the *b1* muscle's oscillations to be lower when the wingbeat amplitude is higher. Our strain measurements support this hypothesis, but our visualizations show that the explanation is incomplete. This is because the lower amplitude oscillations of *b1* on the high-amplitude wing are actually associated with larger oscillations of the basalare sclerite (Figure 7; Movie S3, view here). The picture is further complicated by the fact that *b3*, which is expected to act antagonistically with *b1*, also has higher amplitude oscillations on the high-amplitude wing (Figure 6A).

To resolve this puzzle, we examined the movements of the basalare sclerite in greater depth. Our visualizations show that movement of the basalare sclerite is dominated by rotation about its external head on the low-amplitude wing, but by dorso-ventral translation of the whole sclerite on the high-amplitude wing (Movie S2, view here; Movie S3, view here). Consequently, the internal tip of the basalare sclerite traces an orbit that is aligned with *b1* on the low-amplitude wing, but with *b3* on the high-amplitude wing (Figure 5C; Movie S2, view here). These different modes of oscillation of the basalare sclerite explain why the strain amplitude is higher on the low-amplitude wing for *b1*, but higher on the high-amplitude wing for *b3*. We cannot determine how this is brought about, but one possibility is that the variable stiffness of the *b1* muscle alters the impedance of the system anisotropically. Another possibility is that the orientation of the basalare sclerite is altered by the large *b2* muscle [17],[19], which, like *b1*, has a lower mean strain on the higher amplitude wing (Figure 6D).

Turning manoeuvres are associated with asymmetric aerodynamic power requirements, which cannot be met by varying the output of the power muscles asymmetrically [35]. We hypothesise that changing the mode of oscillation of the basalare sclerite serves to increase the amount of kinetic energy transferred to *b1* on the low-amplitude wing, thereby absorbing excess muscle output. To test the plausibility of this hypothesis, we combined our measurements of *b1* muscle strain with the results of a previous work-loop study [20], to estimate the amount of negative work being done by *b1*. Unlike the other steering muscles, *b1* is typically active on both wings, although it is not necessarily activated on every wingbeat. We estimate that *b1* would have done negative work at a rate of 0.04–0.06 mW on the high-amplitude wing (0.02 mW if inactive) and 0.18–0.30 mW on the low-amplitude wing (0.06 mW if inactive). These intervals bracket the entire range of possible activation phase, and show that the *b1* muscle could have been doing negative work at a rate up to 0.28 mW higher on the low-amplitude wing. This would be sufficient to manage anything up to a 24% asymmetry in the time-averaged aerodynamic power requirements of *Calliphora*, which have been estimated to be 1.58 mW per wing on the downstroke, and 0.81 mW per wing on the upstroke [36]. Our results therefore demonstrate that the *b1* muscles could play a significant role in asymmetric power management, although it remains an open question whether the activation phase of *b1* is controlled appropriately for this function.

**Figure 9:**
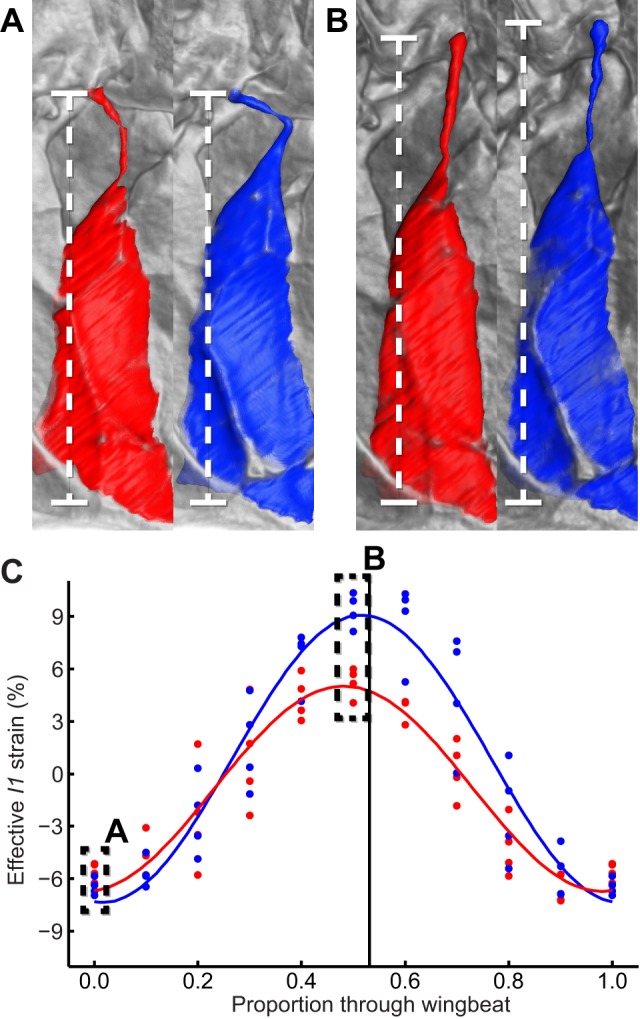
Buckling of the *I1* tendon. (A, B) Visualizations of the *I1* muscle at the start and end of the downstroke, respectively. The tendon is buckled on both wings at the start of the downstroke (A) but has been pulled straight by the end of the downstroke (B). Each panel compares the state of *I1* on the high-amplitude (blue) and low-amplitude (red) wing. (C) effective strain measured along the straight line joining the attachment points of *I1* (dashed line). Comparing the amplitude of this effective strain with the amplitude of the actual *I1* muscle strain in Figure 6F shows that the buckling tendon accommodates a 4-fold enhancement in the range of movement of the first axillary sclerite on the high-amplitude wing.

Our visualizations reveal a completely unexpected behaviour in another steering muscle, showing that the long tendon that connects the *I1* muscle to the first axillary sclerite buckles when the wing is elevated above the wing hinge. This behaviour was observed on both wings in all four individuals, and was always greater on the high-amplitude wing (Figure 9; Movie S2, view here). Buckling only occurs under compressive loading, so it follows that both *I1* muscles must be under compression in the upper part of the wingbeat. Consequently, *I1* contraction cannot possibly increase stroke amplitude by exerting tensile stress on the first axillary sclerite at the top of the upstroke, contrary to what has been inferred previously from static anatomy [6],[7]. Instead, *I1* contraction must limit the movement of the wing at the bottom of the downstroke, thereby reducing stroke amplitude. Consistent with this interpretation, *I1* muscle strain was always lower on the low-amplitude wing. This includes those points in the stroke cycle at which the tendon transitioned between its taut and buckled states. Since the *I1* tendon must have been unloaded at these transition points, the fact that the muscle was shorter on the low-amplitude wing necessarily implies that *I1* must have been contracted on the low-amplitude wing. This conclusion is consistent with the correlations observed in previous electrophysiological studies, which have found that *I1* is only active at reduced stroke amplitude [8]–[10].

Buckling of the *I1* tendon is important for two reasons. First, it accommodates higher amplitude movements of the first axillary sclerite than would otherwise be possible, because the effective strain measured along the straight line joining the origin of the tendon to the origin of *I1* (Figure 9C) has four times the amplitude of the actual strain that the *I1* muscle experiences on the high-amplitude wing (Figure 6E). Second, it means that *I1* contraction will always be intermittent in its effects within each stroke cycle, even if—like *b1*—the *I1* muscle is unable to cycle at wingbeat frequency. Tendon buckling is not unique to *I1*. Although we were unable to visualize the second muscle of the first axillary sclerite (*I2*) fully, our visualizations show that the long tendon of this muscle also buckles on every wingbeat. Tendon buckling also occurs to a lesser extent in *b3* (Movie S2, view here). This previously unknown phenomenon of tendon buckling may therefore be a rather general mechanism in the operation of the blowfly flight motor.

## Conclusions

The fast, complex, three-dimensional movements of the insect flight motor are powered and controlled by several tens of linear actuators, each individually producing only a low-amplitude contractile strain. Here we have presented the first time-resolved visualisations of the workings of this extraordinary mechanism. Our results clearly show that the function of the steering muscles in controlling the wing kinematics can only be understood by placing them in the context of the deforming thoracic structures to which they attach. Deformations of the thoracic wall are not only responsible for transmitting forces from the power muscles to the wings, but are also important in accommodating qualitative changes in the modes of oscillation of the wing articulations. Likewise, deformations of the tendons connecting the steering muscles to the wing articulations are important in accommodating large excursions of the wing articulations, whilst permitting the steering muscles to curtail the wing's movement at certain stages of the stroke cycle. Structural flexibility is known to be important to the aerodynamic efficiency of insect wings [37], and to the function of their indirect power muscles. We have now shown that it is integral also to the operation of the steering muscles, and so to the functional flexibility of the insect flight motor. We anticipate that the insights from this work will inspire the design of future micromechanical systems, and the technique that we have developed is of course applicable to other biological systems exhibiting periodic motion.

## Materials and Methods

### Insect Preparation

Blowflies (*C. vicina*) were collected from a permanent breeding colony at the Department of Bioengineering, Imperial College London and kept on a 24 h (12∶12) light-dark cycle. All individuals were used within two weeks of emergence at ambient lab temperature. Insects were cold-anesthetized at 4°C for 10 minutes and fixed dorsally by the scutum to a wooden tether, using a mixture of beeswax and colophonium. The scutum is a stiff, reinforced thoracic structure [7], and is the standard mounting point for tethered flight preparations in flies. The wooden tethers were attached to a rotation stage using a custom-made holder to align the anteroposterior axis of the animals with the rotational axis of the end station (Figure 1). The insects (*n* = 4) were placed in a 2 ms^−1^ airstream and left to settle into flight for >30 s before recording radiographs.

### Radiograph Acquisition

The X-ray source was a superbending magnet located 25 m from the sample. Monochromatic and polychromatic beam configurations were available, and we ran experiments using both types of configuration for comparison. In the monochromatic configuration (*n* = 2), a double crystal multilayer monochromator was placed 7 m downstream of the source to extract monochromatic X-rays with a bandwidth of 2% at 18 keV photon energy (wavelength  = 0.7 Å) and flux of 8×10^11^ ph/s^−1^ mm^−2^ at the sample site. The monochromator was removed in the polychromatic configuration (*n* = 2), which increased total photon flux by two orders of magnitude and increased the mean photon energy to 35 keV. However, the polychromatic beam was filtered to optimize the bandwidth and the peak wavelength value of the X-rays, which reduced the beam power to an estimated 2×10^12^ ph/s^−1^ mm^−2^ and mainly attenuated longer wavelengths. The beam was 10 mm wide and 4.1 mm high at the sample site under the monochromatic configuration, but was increased in height to 5.7 mm under the polychromatic configuration, which enabled visualization of the entire thorax (Figure 2). The polychromatic beam therefore offers the advantages of a higher flux and larger sampling volume compared to the monochromatic beam, but the algorithms used to reconstruct tomograms from the radiographs assume a specific beam energy, which is better defined for the monochromatic beam. In practice, we found no qualitative difference in the contrast or detail of the radiographs or tomograms between beam configurations, and conclude that both beam configurations allowed comparably good imaging. Results from both configurations are pooled in the analyses which follow.

A 100 μm thick, Ce-doped LuAG scintillator was placed at a distance of 350 mm (monochromatic configuration) or 150 mm (polychromatic configuration) behind the sample to convert the transmitted X-rays into visible light. The scintillator distance was chosen to maximize the phase contrast of the radiographs and was dependent upon the mean photon energy (18 keV for the monochromatic beam and 35 keV for the polychromatic beam). The resulting edge-enhanced image was magnified using a custom-made, high numerical-aperture microscope (Elya solutions, s.r.o) offering continuously adjustable 2- to 4-fold magnification. Projection images were acquired with a pco.Dimax 12-bit CMOS detector system recording at 2,500 Hz for the monochromatic beam and 1,840 Hz for the polychromatic beam, while the insects were rotated at 347° s^−1^ or 332° s^−1^, respectively.

### Stimulus Conditions

The laboratory environment provided a rich, high-contrast, visual scene, which would have stimulated the visual system of the insects strongly during rotation. The rotation rates of 347° s^−1^ and 332° s^−1^ were an order of magnitude higher than the lowest rates known to induce visually stimulated turning reactions in Diptera [9],[14]. The angular velocity of the insect during rotation was three orders of magnitude lower than the mean wingtip velocity, so any bilateral asymmetries in the wing kinematics must have been due to changes in flight motor output in response to the roll stimulus, rather than passive aerodynamic effects due to rotation.

### Measurement of Wingtip Kinematics

Two synchronized Photron SA3 cameras (Photron Ltd) with 180 mm Sigma macro lenses were used to film the blowflies, recording at 4,000 Hz with a 33.3 μs exposure time and at 448×384 pixel image size (Figure 1). Illumination for the cameras was provided by a custom-built infrared LED light source directed onto white card below the insect. The cameras were calibrated using fully-automated calibration software running in Matlab (The Mathworks Inc.) [31]. We tracked the wingtips using background subtraction and manual thresholding to isolate the outlines of the wings in each camera view. The tip of each wing was determined as the point along the outline that was furthest from the wing hinge. The three-dimensional coordinates of the wingtip were then calculated using the camera calibration parameters.

### Grouping of Radiographs by Wingbeat Phase

A data acquisition module (National Instruments USB-6211 DAQ), sampling at 80 kHz, was used to record the exposure times of the Photron SA3 cameras and the pco.Dimax detector system for the purposes of grouping the radiographs. The flies had a mean wingbeat frequency of 145 Hz, so each 4 s recording consisted of approximately 600 wingbeats (Figure 3). We used the measured wingtip kinematics to group radiographs taken from different angles but at identical phases of the wingbeat. We identified the beginning and end of each wingbeat from the wingtip kinematics, and selected the radiographs closest in exposure time to ten evenly spaced phases of each wingbeat for analysis. This allowed us to combine data from all of the wingbeats measured for a given fly, despite the fact that their period was somewhat variable (Figure 3). Our tomographic reconstruction technique therefore produced one composite wingbeat for each individual, comprising ten time steps, where every time step pools radiographs from c. 600 wingbeats.

### Tomographic Reconstruction

The projections were despeckled to remove bright pixels caused by scattered X-rays hitting the detector, and were flat field corrected with the average flat-beam images (i.e., images taken with no sample) and dark images (i.e., images taken with no beam) acquired immediately after the scan. Phase retrieval was performed in a qualitative manner using the ANKAPhase implementation [38] single image phase retrieval algorithm under the assumption that the object consisted of a homogeneous soft tissue material [39]. We assumed that the steering muscles had a refractive index equal to that of water [40]. For the monochromatic beam, the real and imaginary parts of the deviation from one of the complex refractive index of the material were 7×10^−7^ and 5×10^−10^, respectively. For the polychromatic beam, we assumed that the mean X-ray energy was 35 keV and used values of 2×10^−7^ and 10^−10^ for the real and imaginary parts, respectively, of the decrement from one of the index of refraction. Tomographic reconstruction was performed using a Fourier transform-based algorithm [41]. The resulting voxels had an isotropic spacing of 3.3 μm, with no discernible difference between tomograms collected using the monochromatic or polychromatic beam.

### Measurements of Muscle Kinematics

**Figure 10:**
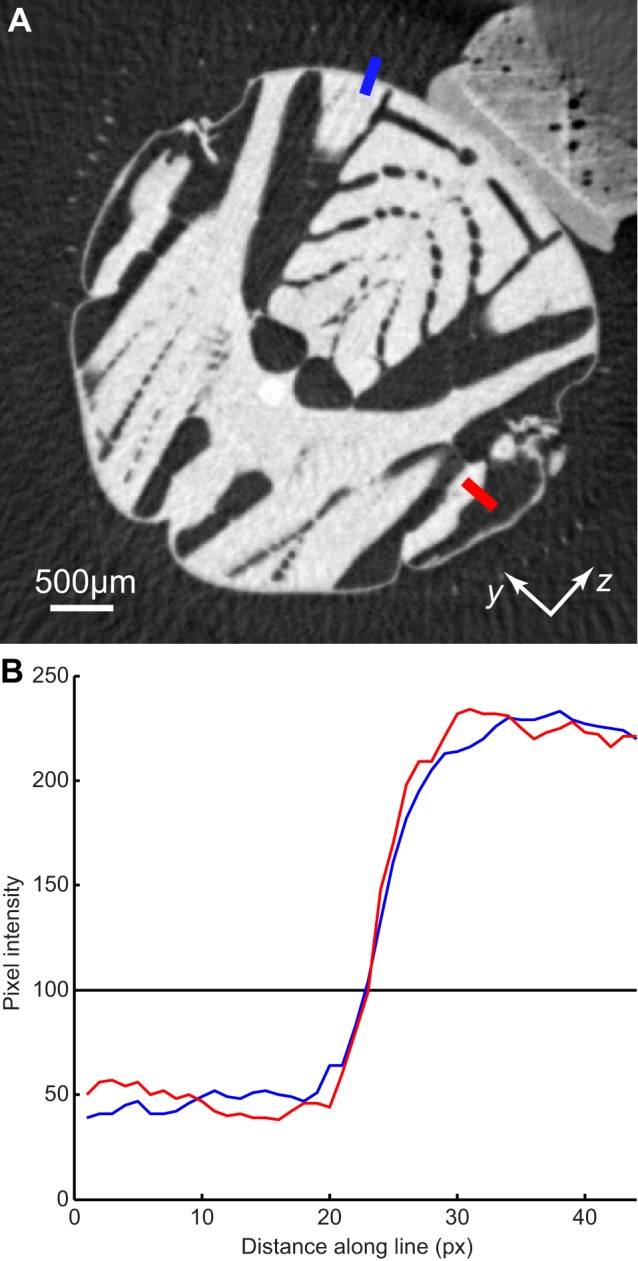
Edge detail of two parts of the thorax. (A) Tomogram showing transverse section of the thorax, with the mount visible in the upper right of the image. The blue line cuts through the scutum, which is a rigid part of the thorax that did not move measurably during recordings. The red line cuts through the steering muscles, which oscillate at wingbeat frequency. (B) Pixel intensities along the two lines indicated in (A). Edge sharpness, as measured by the steepness of the change in pixel intensity along each line, is essentially identical for the scutum and the steering muscles. This indicates that the position of the steering muscles must have been consistent between wingbeats, at the phase of the wingbeat shown here, which indicates that the steering muscle kinematics did not vary measurably between wingbeats.

The tomographic data were visualized and segmented using Amira (VSG). We segmented the data using a manual threshold that separated the muscles and cuticle from the surrounding material (Figure 5). The manual threshold was chosen at a level approximately double that of the background noise (Figure 10). The end points of the muscles were manually tracked using natural features as markers to ensure that the same parts of the muscles were tracked from one frame to the next and between individuals (Figure 5A). These end points were then used to calculate the lengths for each steering muscle (Figure 5B). Both *b3* and *I1* exhibited tendon buckling during parts of the wingbeat. To take account of this, we used three-dimensional skeletonization [42] to find the line running through the centre of the tendon, which was then connected to the muscle ends to form a continuous line (Figure 5B).

### Statistical Analysis

A sinusoid of arbitrary mean, amplitude, and phase can be expressed as a linear combination of a sine function, a cosine function, and a constant. For each pair of steering muscles, we used a single linear model to regress the strains that we had measured for both wings on the sine and cosine of the wingbeat phase, comparing the fitted coefficients between wings. We did not control separately for fly identity, because the strain measurements had already been normalized by the mean value for each fly, such that the mean strain was the same for all flies (i.e., equal to zero). We used a Monte Carlo method to transform the 95% confidence intervals for the parameter estimates of the linear model into 95% confidence intervals for the mean, amplitude, and phase of the strain oscillations. This allowed us to test statistically for differences in the mean, amplitude, and phase of the strain oscillations between the high- and low-amplitude wings (Figure 8).

### Estimation of Negative Work Done by the b1 Muscles

Tu and Dickinson [20] measured the negative work done by the *b1* muscle at different amplitudes of oscillatory strain, and with different phases of muscle activation, using the work loop technique. We interpolated their data to estimate the range of negative work that would be done by the muscle with the measured strain amplitudes of 2.3% and 5.5%. This allowed us to estimate that the net negative work done per wingbeat would have been in the range 0.25–0.41 μJ for the high-amplitude wing, and in the range 1.23–2.06 μJ for the low-amplitude wing, depending upon the unknown phase of muscle activation. The mean wingbeat frequency in our data was 145 Hz, so the *b1* muscle would have been absorbing kinetic energy at a rate of 0.04–0.06 mW on the high-amplitude wing and 0.18–0.30 mW on the low-amplitude wing. If the muscle were inactive on either the high- or low-amplitude wing, kinetic energy would have been absorbed at a rate of 0.02 mW and 0.06 mW, respectively.

### Effects of Tethering and Radiation Exposure

Tethering is known to affect wing kinematics in other dipteran species [43], but there is a paucity of free-flight data for *Calliphora* with which to compare our tethered wing kinematics, particularly during the roll manoeuvres that we have simulated. The mean wingbeat frequency (145±11 Hz) and mean stroke plane angle on each wing (46.8°±4.1° low-amplitude wing, 68.0±9.6° high-amplitude wing), were within ranges observed in a free-flying *Calliphora* [44], with similar wing length (9.2±0.5 mm free-flight data versus 8.7±0.4 mm in our data). Mean stroke amplitude on the high-amplitude wing (141°±7°) was also within the range of free-flying *Calliphora* (123°–150°), but the mean stroke amplitude on the low-amplitude wing (100°±9°) was slightly lower than previously recorded. However, free-flight kinematics have only been measured in symmetric flight conditions, and *Calliphora* typically reduce the stroke amplitude on the ipsilateral side during roll manoeuvres, rather than increasing it on the contralateral side, consistent with our measured kinematics [32]. Thus, we cannot discount an effect of tethering on our insects, but their wing kinematics appear to be broadly representative of those used during free-flight.

A concern with using high-power X-rays to examine the biomechanics of the insect flight motor is that the radiation may affect the physiology of the insects during recording [45]. All four individuals continued flying after recording stopped, but although their measured wing kinematics fluctuated during recordings, there was no systematic change in the wing kinematics over the recording period (Figure 3). Stroke amplitude was bilaterally asymmetric throughout each recording, and was consistent with the asymmetry expected during a compensatory roll response, indicating that the flies were responsive throughout to the roll stimulus that we provided.

Further evidence of the consistency of the flies' behaviour is provided by the quality of the tomograms themselves, because the tomographic reconstruction process will only be successful if the pose of the sample is consistent within each group of radiographs. Any significant variation in steering muscle kinematics between wingbeats would result in blurring of the reconstructed tomograms, which each represent the average state of the flight motor at a given phase of the wingbeat. The edge detail of the rigid scutum had similar edge sharpness to the steering muscles (Figure 10), which indicates that the steering muscle kinematics were consistent through each recording.

Notwithstanding the consistency of their wing and muscle kinematics during the recordings, and the fact that the flies continued to fly immediately following exposure, all four individuals died a short while after. We therefore calculated the radiation dose received by the flies to assess the severity of exposure. Most of the X-rays produced by the beamline pass through the insects, but the amount will be dependent on both the individual (due to variation in size and hydration) and beam energy. We determined the proportion of X-rays absorbed by the insects by measuring the difference in image intensity between flat-beam images and radiographs where the insect was in the beam, using a region of interest containing the thorax, but not the mount. Using this method, we estimated that the mean absorption was 23% for the monochromatic beam and 13% for the polychromatic beam. The absorbed dose (*D*) was calculated as the absorbed power per unit mass:
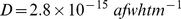
where *a* is the proportion of the beam absorbed by the insect, *f* is the beam flux, *w* is the width of the insect exposed to the beam (estimated from the radiographs to be 3.3 mm), *h* is the height of the beam, *m* is the mass of the insect (assumed to be 82 mg [20]), and *t* is the recording duration (4 s). The estimated total dose was 350 Gy for the monochromatic beam and 1,300 Gy for the polychromatic beam.

These total doses are similar to or less than the doses that have been applied to other insects in previous work without any measurable long-term effect [45]. However, our dose rates (90 Gy s^−1^ and 325 Gy s^−1^, for the monochromatic and polychromatic beam, respectively) were at least an order of magnitude higher than those used in previous work [45]. We therefore attribute the adverse effects of radiation following exposure to the high rate at which the dose was supplied.

## Supporting Information

Movie S1 **Three-dimensional visualization of the insect thorax.** This video shows the insect thorax reconstructed from tomograms and highlights the external movements of the thorax and the location of the indirect power and steering muscles. This video can be viewed at http://youtu.be/P6lBkK3J9wg.
(MOV)

Movie S2 **Three-dimensional visualizations of five of the direct steering muscles.** The muscles are shown for the high-amplitude (left) and low-amplitude (right) wings through ten stages of the wingbeat, starting at the beginning of the downstroke. The steering muscles are viewed from the inside of the thorax looking out toward the wing hinge, and other parts of the thorax have been removed for clarity. The view of the low-amplitude (right) wing muscles has been mirrored about the sagittal plane of the insect for ease of comparison. The basalare sclerite is not visible directly in the reconstruction, but its position can be inferred by the intersection of the *b1* and *b3* steering muscles. See main text and Figure 6 for labeling of muscles. This video can be viewed at http://youtu.be/ehG4G-NOTQg.
(MOV)

Movie S3 **Three-dimensional visualizations of the external movement of the thorax.** Differences in the deformations of the thorax and the movement of the basalare sclerite are shown for the high-amplitude (left) and low-amplitude (right) wings through ten stages of the wingbeat, starting at the beginning of the downstroke. The low-amplitude (right) view has been mirrored about the sagittal plane of the insect for ease of comparison. See main text and Figure 7 for anatomical details. This video can be viewed at https://www.youtube.com/watch?v=Cxc3yZIsbqo&feature=youtu.be.
(MOV)
